# Person-Centered Care Planning for People Living With or at Risk for Multiple Chronic Conditions

**DOI:** 10.1001/jamanetworkopen.2024.39851

**Published:** 2024-10-17

**Authors:** Brittany N. Watson, Lilly Estenson, Aimee R. Eden, Maya T. Gerstein, Maria Torroella Carney, Vonetta M. Dotson, Trisha Milnes, Arlene S. Bierman

**Affiliations:** 1 Department of Family and Community Medicine, Wake Forest University School of Medicine, Atrium Health Wake Forest Baptist, Winston-Salem, North Carolina; 2 Leonard Davis School of Gerontology, University of Southern California, Los Angeles; 3 Agency for Healthcare Research and Quality, Rockville, Maryland; 4 Department of Medicine, Division of Geriatrics and Palliative Medicine, Zucker School of Medicine at Hofstra/Northwell, Northwell Health, New Hyde Park, New York; 5 Department of Psychology, Georgia State University, Atlanta; 6 Gerontology Institute, Georgia State University, Atlanta; 7 Charlie Norwood VA Medical Center, Augusta, Georgia

## Abstract

**Importance:**

The US has a growing population of people living with multiple chronic conditions (MCC), yet the health system is ill designed to meet their needs. Person-centered care planning (PCCP) is an approach to provide comprehensive care that is responsive to the individual to improve health outcomes and increase value.

**Objective:**

To examine strategies used to provide PCCP for people living with or at risk for MCC, as well as facilitators and barriers to implementation.

**Design, Setting, and Participants:**

This qualitative study uses thematic analysis of responses from a Request for Information (RFI) published in the Federal Register soliciting input on PCCP, posted by the Agency for Healthcare Research and Quality (AHRQ) in 2022. The RFI was available for public comment from September 16, 2022, to November 15, 2022. Responses were analyzed between January 2023 and February 2024. Respondents were individuals and organizations who identify as, provide care for, or seek to improve care for individuals living with or at risk for MCC. Some responses represent multiple individuals.

**Main Outcomes and Measures:**

Qualitative themes and subthemes of the RFI responses, developed using thematic analysis through inductive and deductive coding of the open-text responses.

**Results:**

There were a total of 58 respondents, including clinicians, researchers, patients, caregivers, and representatives from health care payer, practitioner, health system, advocacy, professional, and supporting nonprofit and industry organizations. Researchers identified 9 themes: (1) suboptimal quality of care; (2) person-centered, goal-concordant care; (3) multidisciplinary team–based care and care coordination; (4) prevention across the life course; (5) digital health solutions; (6) workflow; (7) education and self-management support; (8) payment; and (9) achieving community, health system, and payer goals.

**Conclusions and Relevance:**

In this qualitative study of PCCP for people living with or at risk for MCC, challenges to widescale adoption of PCCP were identified along with strategies to address these challenges, including the alignment of payment, policy support, culture change, adoption of meaningful measures, and the need for evidence on strategies to scale and spread PCCP. Insights gained from this analysis can inform research priorities and implementation efforts to advance PCCP as an integral component of routine care.

## Key Points

**Question:**

What do persons seeking care and their caregivers, health care practitioners, professional and nonprofit associations, and health care organizations believe are innovative approaches and challenges to implementing person-centered care planning (PCCP) for people living with or at risk for multiple chronic conditions?

**Findings:**

In this qualitative study including 58 respondents, 9 themes were identified through a thematic analysis of responses to the Agency for Healthcare Research and Quality’s Request for Information. Themes described key components of PCCP for multiple chronic conditions and challenges and strategies to overcome them, including multidisciplinary teams, digital health technologies, workflow changes, payment policies, and goal alignment.

**Meaning:**

These findings suggest that despite barriers to implementing PCCP, there is much opportunity to transform care delivery by amplifying and coordinating current efforts.

## Teaser

This qualitative study describes opinions, strategies, and facilitators and barriers to implementation of person-centered care planning from individuals and organizations who identify as, provide care for, or seek to improve care for individuals living with or at risk for multiple chronic conditions.

## Introduction

People living with multiple chronic conditions (MCC), the most common chronic condition seen in clinical practice, often receive fragmented care focused on treating multiple individual conditions rather than whole-person care consistent with their priorities and circumstances, resulting in suboptimal health outcomes and avoidable adverse events.^1,2^ There is an urgent need to improve care delivery for this diverse, heterogeneous, growing, and high-cost population. Through extensive stakeholder consultation and literature review, the Agency for Healthcare Research and Quality (AHRQ) identified person-centered care planning (PCCP) as central to improving the quality and outcomes of MCC care. PCCP, the process of developing a holistic plan of care addressing the needs of the whole person in the context of their life, may occur through team-based collaboration between clinicians, patients, and patients’ broader care networks toward the coordinated identification, treatment, and management of physical and behavioral health conditions, while also addressing social determinants of health.^3,4,5^ Patients engage in shared decision-making with clinicians and care team members to develop an individualized care plan aligned with their preferences and goals.^6,7,8^

Comprehensive, longitudinal PCCP can improve health care quality and value for people living with or at risk for MCC by decreasing care fragmentation, increasing access to preventive services,^9,10,11^ reducing unnecessary hospital and emergency service utilization,^12,13^ and improving patient experience.^11^ While organizations that provide certain Medicare and Medicaid postacute and long-term care services are statutorily required to create care plans^14,15^ and care plans have been required in Medicaid managed care and some Centers for Medicare & Medicaid Innovation (CMMI) demonstrations,^16,17,18^ barriers to its routine use in practice are many.^19^

Clinicians and health care organizations are developing innovative approaches to providing PCCP to patients with MCC, overcoming barriers to implementation. However, their experience is not always published in the academic or gray literature. To inform its programmatic work and that of its federal partners, AHRQ released a Request for Information (RFI) in the Federal Register on PCCP for MCC to solicit comment on the current state of comprehensive, longitudinal PCCP for people at risk for or living with MCC across care settings (eg, health systems, primary and other ambulatory care, hospitals, home care), including existing models of PCCP and barriers and facilitators to implementation.^20^ The RFI also sought comment on innovative models of care, approaches, promising strategies, and solutions to enable clinicians and practices to routinely engage in PCCP.^20^ We qualitatively analyzed these responses to inform future work and support the increased uptake and scale of PCCP.

## Methods

This qualitative study was determined to be exempt from human participants review and informed consent by AHRQ’s Human Protections Administrator because it does not constitute human participants research as defined by 45 CFR 46. This report aligns with the Standards for Reporting Qualitative Research (SRQR) reporting guideline.

The RFI was posted in the Federal Register on September 16, 2022. It included 27 questions developed by AHRQ with input from other agencies within the Department of Health and Human Services.^20^ The RFI announcement was shared widely via AHRQ and partnering agencies’ email listservs and websites, as well as the websites of private sector organizations. Representatives of diverse perspectives, including persons living with MCC and their caregivers, clinicians and other health care workers, professional societies, health systems, payers, human service and community organizations, and information technology professionals and vendors were encouraged to respond to the RFI via email by November 15, 2022. Respondents were instructed to answer questions that were applicable to their work or experience, and to contribute any additional relevant information that was not directly addressed in the RFI.

Our research team, including health services researchers with qualitative and quantitative expertise and clinicians specialized in primary care and aging, conducted a qualitative thematic analysis of open-text responses to identify prominent topical categories and overarching themes. Our process, modified from the framework by Braun and Clarke,^21,22,23^ included data familiarization, inductive and deductive coding, theme generation, theme development, theme consensus, and reporting findings.^21,22,23^ This method is consistent with our goal to increase understanding of PCCP implementation to inform policy and practice.^22,24,25^ After initial data familiarization, 1 author (B.W.) coded each response using codes identified a priori as well as new, open codes derived from the response data. Additional authors (A.B., A.E., and L.E.) created memorandums to document their observations, compare preliminary coding, and identify further codes. We refined the codebook in team meetings held throughout the coding process, adding definitions and sample quotes to further specify code meanings and reach agreement on code assignments. We then organized codes and corresponding quotations into topical and conceptual themes and subthemes. Using this complementary inductive/deductive approach ensured that codes and themes were informed by but not restricted to the content of the RFI questions. All authors were involved in discussions to develop, define, and refine the final themes and subthemes and to ensure accurate application to respondent quotes. This iterative process included more than 15 rounds of collective theme review and revision. We used NVivo software version 12.0 (QSR International), Excel spreadsheets version 2308 (Microsoft), and Word documents version 2308 (Microsoft) in the analysis. Responses were analyzed between January 2023 and February 2024.

## Results

A total of 58 respondents, including clinicians, researchers, patients, caregivers, and representatives from health care payer, practitioner, advocacy, professional, and supporting nonprofit and industry organizations responded to the RFI (Table 1). Respondents represented diverse populations, including older adults, adults with disabilities, caregivers of children with medical complexities, and low-income families. Aggregated responses totaled approximately 87 000 words, with variation in length and format among responses. No respondent answered all 27 questions; respondents addressed the questions that were most relevant to them and their expertise and/or provided comment. We identified 9 themes and 52 subthemes. Themes with definitions and subthemes are listed in the Box and subthemes with representative quotes are presented in Table 2.

**Table 1.** Stakeholder Groups Represented by Request for Information Respondents

Respondent	No. (N = 69)^a^
Individuals	
Clinician^b^	15
Researcher or academic	11
Patient, family, or caregiver	5
Health educator	4
Total	35
Organizations	
Health system or practice	6
Nonprofit quality improvement, support, or advocacy organization^c^	15
Industry^d^	3
Payer	2
Professional association	8
Total	34
Total	69

^a^ Of the 58 RFI respondents, 11 were counted twice because they represent 2 stakeholder groups: clinician and researcher/academic (10 respondents) and health system or practice and payer (1 respondent).

^b^ Clinicians include physicians, nurse practitioners, nurses, social workers, and occupational therapists.

^c^ Nonprofit quality improvement, support, and advocacy organizations include health policy think tanks, quality accreditation organizations, and patient advocacy organizations.

^d^ Industry representation includes consulting firms and health information technology companies.

Box. **Themes, Theme Definitions, and Subthemes**

**Theme 1: Suboptimal Quality of Care**

Motivation for implementing person-centered care. These are systemic issues in the health care system that person-centered care planning aims to remedy.

1. Impaired access to care and needed services
2. Inadequate treatment
3. Care fragmentation
4. Suboptimal health outcomes
5. Avoidable acute care utilization
6. Inadequate practice improvement support

**Theme 2: Person-Centered, Goal-Concordant Care**

Care that is individualized and meets patients, families, caregivers, and communities where they are, both physically and figuratively, to maximize patient access, autonomy, and engagement and to align care delivery with a person’s goals, preferences, and social context.

1. Goal-concordant care
2. Shared decision-making
3. Facilitating access to information and services
4. Addressing social needs
5. Addressing language and cultural barriers
6. Health systems culture change
7. Insufficient evidence
8. Home- and community-based care and services

**Theme 3: Multidisciplinary Team-Based Care and Care Coordination**

The composition and structure of multidisciplinary care teams. The coordination or integration of health and social care across care settings (eg, postacute care to home) and care disciplines requires communication and collaboration between care team members and care organizations. Sometimes used interchangeably with shared care, care management, or case management, although these terms can also have distinct meanings.

1. Multidisciplinary team approach
2. Investment in the health care workforce
3. Increasing health care practitioner and team competencies
4. Including individuals with lived experience on the team
5. Behavioral health integration
6. Collaboration across specialties
7. Linkage to social and community resources
8. Care transitions

**Theme 4: Prevention Across the Life Course**

The prevention of MCC and functional decline as well as the prevention of chronic disease progression, harm, and injury among individuals at risk through primary, secondary, and tertiary prevention.

1. Life course perspective to prevention
2. Primary prevention
3. Prevention of disease progression and functional decline
4. Moving from a disease-centered focus to a person-centered focus

**Theme 5: Digital Health Solutions**

The transfer of information between patient and clinician throughout the patient journey, including ethical and intelligent use of patient data for clinical care, quality improvement, and research.

1. Prioritizing user experience
2. Use of shared electronic care plans
3. Interoperability and data integration
4. Patient and caregiver access to EHR data
5. Evidence-informed decision tools
6. Remote digital monitoring

**Theme 6: Workflow**

The sequence of events and process of care planning. This includes tools, practices, modalities, and technologies used in care planning by care team members.

1. Redesigning workflow to enable person-centered care for patients with MCC
2. Workflow enables dynamic comprehensive shared care planning
3. Using technology to foster collaboration (shared care)
4. Time pressures
5. Hybrid (in-person and virtual) care delivery models to engage patients
6. Risk stratification to allocate time and resources based on need

**Theme 7: Education and Self-Management Support**

The need to increase awareness among patients and clinicians to become comfortable with the concepts and implementation of person-centered care planning. Establishing supportive programs, protocols, resources, and tools that allow patients and caregivers greater direct involvement and control over the management of their conditions. Activities could include education, training, and shadowing.

1. Increase patient knowledge about the role and benefits of comprehensive care planning
2. Patient and caregiver education
3. Clinician education
4. Empowering patients to self-manage and self-advocate

**Theme 8: Payment**

How health care practitioners are compensated for the care they provide to people living with MCC.

1. Address insufficient and misaligned payment
2. Increase value by improving quality and outcomes
3. Adopt value-based payment models that include prevention or slowing of functional decline
4. Pay for interdisciplinary teams
5. Fund integrated person-centered care models
6. Eliminate out-of-pocket costs for care planning and management services

**Theme 9: Achieving Community, Health System, and Payer Goals**

Community, health system, and payer goals for successfully implementing person-centered care planning within primary care and other ambulatory settings.

1. Address inequities in resource distribution
2. Develop meaningful measures that assess quality of person-centered care
3. Harmonize and align measures to drive improvement
4. Demonstrate and document return on investment

Abbreviations: EHR, electronic health record; MCC multiple chronic conditions.

**Table 2.** Themes and Subthemes with Representative Quotes

Theme	Relevant quotation	Respondent
**Theme 1: Suboptimal quality of care**		
1.1: Impaired access to care and needed services	“When doing comprehensive planning with a seriously at-risk person, the process is constrained mostly by what is reasonably available to this person in this situation. The patient and care team might benefit from home-delivered food; but, if there is a 6-month waiting list in this community, having home-delivered food is not available to the planning process. If there is no disability-adapted housing available in this community, the patient and care team may have to look to facility-based care.”	Clinician and researcher/academic
1.2: Inadequate treatment	“I have multiple chronic debilitating health conditions and am forced to live in pain daily… I physically appear to the eye to look ok and that puts me even at more disadvantage. I feel that this fact alone causes me to be [minimized] in my care by what little staff that is available to us in this area.”	Patient, family, or caregiver
	“Patient defined psychosocial priorities are less likely to be addressed than medical priorities due to the primary care provider’s need to address acute and chronic medical concerns, symptoms, questions, referrals and test results in limited time. According to the National Alliance on Mental Illness (NAMI), the average delay between mental health symptoms and treatment is 11 years.”^a^	Professional association
1.3: Care fragmentation	“Individuals with multiple chronic conditions… often receive fragmented, uncoordinated, and crisis-driven care, with no strong evidence base guiding their management and treatment.”	Professional association
1.4: Suboptimal health outcomes	“Individuals with multiple chronic conditions (MCCs) require intensive and specialized clinical care provided by an array of health care professionals…Because of these complex needs, individuals with MCC are vulnerable to suboptimal outcomes resulting from access, continuity, fragmentation, and quality problems found within and between clinical, social, and community service systems.”	Payer
1.5: Avoidable acute care utilization	“[Care] fragmentation leads to excessive utilization of expensive health care resources, including emergency department visits, hospitalizations, and suboptimal health outcomes.”	Professional association
	“There is overwhelming evidence that the population is ageing and that the current fallback position for the elderly who are naturally and normally dying to be admitted to acute hospitals where little can be offered for chronic health conditions apart from fine tuning which could be achieved by community health services.”	Clinician and researcher/academic
1.6: Inadequate practice improvement support	“At the outset we stress that our country urgently needs a national health care system that is much better at delivering proven effective evidence-based treatments to patients at the primary point of care. It is unacceptable that it currently takes 17 years to translate research findings to clinical care.”	Nonprofit quality improvement, support, or advocacy organization
	“I have come to realize that Family Practice providers must medically know everything about everything, and have much more to oversee, as opposed to a specialty provider. In my opinion, Family Practice providers need to be paid better because they must continually educate to keep up on all new medical knowledge, in all arenas, and navigate many conditions (such as Multiple Chronic Conditions) in every visit, which is far more time consuming and labor intensive.”^a^	Clinician
**Theme 2: Person-centered and goal concordant care**		
2.1: Goal-concordant care	“The American Geriatrics Society developed a beautiful definition of person-centered care: ‘Person-centered care means that individuals’ values and preferences are elicited, and once expressed, guide all aspects of their health care, supporting their realistic health and life goals.’”^19^	Clinician and researcher/academic
	“A key principle of person-centered care is prioritization, focusing on the strategies most likely to help a patient achieve their goals.”	Clinician and researcher/academic
2.2: Shared decision-making	“Clinical care teams must work collaboratively with patients, families, and caregivers to empower them to be active partners in all aspects of care. Patient and family needs, preferences, and limitations should be actively anticipated, solicited, and considered in all care encounters and decisions. Patients and families should additionally feel empowered to be active partners in their own care but should not be responsible for the coordination of care between their PC and SC teams. Clinical teams should also incorporate patient input into all medical decision making, including but not limited to those about initial specialty care referrals and ongoing engagement. Clinicians engaging one another, the patient/caregiver, and the family in shared decision making is critical to achieving positive health outcomes.”	Professional association
	“The ability to share comprehensive data, including behavioral health data, is important for whole person care. Patient-expressed goals and being able to share this [data] is important for engaging patients in shared decision making.”	Industry
2.3: Facilitating access to information and services	“For people experiencing homelessness, we have created multiple Primary Care Safety Net (PCSN) practices across [our health system] to broaden access to this specialized care setting. Within the clinics, we have redesigned the way we provide primary care. Our medical providers’ schedules have protected time to accommodate same-day, walk-in appointments alongside scheduled patient visits that don’t carry penalties for late arrivals. Appointments are longer for both new and established patients so that there is adequate time to develop a trusting, therapeutic relationship and manage complex health and social issues. And to help patients navigate housing, social issues, and medically complex care plans, there are embedded community health workers who have training in housing navigation, work with patients for extended periods of time, and are in frequent contact with their clients to ensure they have someone to call on for support with needs both big and small.”^a^	Health system or practice
2.4: Addressing social needs	“We screen our [plan] members for the following core HRSNs: financial strain, food insecurity, housing insecurity, loneliness, and transportation barriers. These five HRSNs… were chosen based on several factors, including: prevalence within the populations we serve, association with unanticipated health care utilization among [the] population, and their impact on health-related quality of life.”	Health system or practice and payer
2.5: Addressing language and cultural barriers	“Linguistic competence plays a significant role in the acceptance of cultural constructs and can be defined as the provision of culturally appropriate oral and written language services. To implement programmatic strategies to address existing cultural constructs, their existence must not only be accepted but also understood.”	Nonprofit quality improvement, support, or advocacy organization
2.6: Health systems culture change	“Moving from problem-oriented care to person-centered care requires a major shift in thinking (mindset). Most health care professionals, and particularly physicians are trained to focus on strategies and objectives rather than patient-relevant goals (eg, length and quality of life, personal growth and development, a good death).”	Clinician and researcher/academic
2.7: Insufficient evidence	“Care planning for people living with multiple chronic conditions is complex and the evidence to support decision-making is often not available.”	Industry
2.8: Home- and community-based care and services	“IDT members practice at the [program’s] center and in participants’ homes, and participants may receive primary care, behavioral health, oral care, therapy, meals, recreation, socialization, and personal care integrated with long term care services and supports in either setting.”	Nonprofit quality improvement, support, or advocacy organization
**Theme 3: Multidisciplinary team-based care and care coordination**		
3.1: Multidisciplinary team approach	“The solution [for implementing programs for complex patients using person-centered care planning] is multidisciplinary and multifaceted. Operationally, this may involve providing involved providers and their teams with appropriate space and staffing, including clinician, nurse, social worker, and CHW/wellness advocate to provide a comprehensive, integrative approach to complex households.”^a^	Clinician and researcher/academic
3.2: Investment in the health care workforce	“…resources must be present to both encourage and facilitate more frequent contact in the community between patients and their care team…. This would of course require a massive investment in jobs, including (and perhaps especially) in the Home Care space.”	Health system or practice
3.3: Increasing clinician and team competencies	“Further, dedicated care management personnel (i.e., discharge coordinators, home health department, utilization review specialists, care management specialists) who can coordinate emergency department (ED) visits, post-acute care, telemedicine follow ups, medication refills, and even some insurance claims issues, are of paramount importance and need to be supported through adequate training.”	Professional association
3.4: Including individuals with lived experience on the team	“The terms critical to successful care planning and maximal outcomes in face of multiple chronic conditions are patient, caregiver (including family), patient advocate, and advocacy.”	Nonprofit quality improvement, support, or advocacy organization
	“Leveraging staff with lived experience can be a particularly important strategy for addressing social needs and advancing health equity as these individuals are generally from the same communities as the patient they serve, leverage lived experience to engage patients and built [sic] trusting relationships and have deep knowledge of local resources.”	Nonprofit quality improvement, support, or advocacy organization
3.5: Behavioral health integration	“Primary Care Behavioral Health (PCBH) is a model which integrates behavioral health into primary care by embedding behavioral health providers as part of the primary care team. The behavioral health providers not only help to identify issues which impact health, they are able to provide brief interventions tailored to patient and provider goals. PCBH allows issues to be identified and treated earlier and can help to prevent a decade of suffering and disability.”^a^	Professional association
3.6: Collaboration across specialties	“It is important to note that we are not doing this work in isolation as a healthcare system—we are actively partnering with the Department of Homeless Services, Correctional Health Services, Department of Health and Mental Hygiene, as well as established community-based organizations. Open communication and careful coordination ensure that we are able to deliver a cohesive care experience for our patients.”	Health system or practice
	“PC and SC clinicians who share patients simply cannot work in silos. It is critical that all clinicians have a shared understanding of each other’s role in care[…] Shared expectations should include: [1] The primary care team serving as the ‘hub’ or central organizer of a patient’s overall care. [2] A collaborative approach between PC, SC, and patients/caregivers which determines the appropriate level of specialty care involvement and changes over time. [3] An understanding by each party of what the specialty role entails, and which clinician and care team is responsible for what aspects of care… Having common, agreed-upon principles and expectations for who does what in each type of care relationship establishes a foundation for reducing ambiguity and providing coordinated care for the patient.”	Professional association
3.7: Linkage to social and community resources	“There needs to be robust evaluation and intervention of social determinants of health. For example, it’s not useful to put things into a care plan relating to social determinants of health that a patient can’t execute, such as in the areas of housing, food insecurity, etc. Research to date has focused on the tool (care plan) rather than on making the connections needed to provide the communication and care. The document isn’t the intervention.”	Professional association
	“Outside of clinical settings, health care systems can partner with social service agencies and community-based organizations to engage, screen, and exchange SDOH information for shared service users.”	Payer
3.8: Care transitions	“A crucial step in this process is the creation of a transition plan, and related documents, with the patient/family during an office/clinic visit devoted to the transition of care. [The] High Value Care Coordination (HVCC) tool kit defines these expectations and provides critical elements for a high value referral process.”	Professional association
	“Care transitions are an inflection point where a full accounting of a patient’s treatment, functional, and social needs are necessary to optimally plan and execute the next phase of care.”	Payer
**Theme 4: Prevention across the life course**		
4.1: Life course perspective to prevention	“Ensuring that pre-K, K-12, and employers are on board with MCC prevention will help to mitigate the health of most students and employees…It is much better to do the hard work up front than to try and clean up on the back end.”	Clinician
4.2: Primary prevention	“In all of these evidence based programs that I have led, I see in many [patients] that light bulb that helps them to understand their health is in their hands and through our support and the participants in the class, there is those small behavior changes that lead to the bigger health goals. It can give them hope which we all need from the trials of the [COVID-19] pandemic. To support these classes makes economically [sic] sense. If we reach just one person a year that will not be visiting the ER multiple times and losing their independence too soon, [it] makes up the money spent on the programs.”	Health educator
4.3: Prevention of disease progression and functional decline	“Occupational therapy practitioners use performance-based assessments and address barriers to function through focused evidence-based interventions that remediate or restore abilities, enhance existing skills, create opportunities, and promote wellness to prevent relapse through modifying or adapting the environment or activity to help people with MCC achieve full participation in the everyday activities that are meaningful to them.”	Professional association
4.4: Moving from a disease-centered focus to a person-centered focus	“Person-centered care is conceptually different from disease-oriented care. The focus is on life goals rather than on disease prevention and management, which are viewed as obstacles, challenges, or opportunities.”	Clinician and researcher/academic
**Theme 5: Digital health solutions**		
5.1: Prioritizing user experience	“Single sign-on for patients and designated family/caregivers with flexible modalities (e.g., smart phone/computer, remote patient monitoring) can more fully engage individuals in their care.”	Nonprofit quality improvement, support, or advocacy organization
	“The importance of data collection and informatics cannot be underestimated.… A barrier to continuity in documentation is the lack of a standard electronic medical record (EMR). Having a standard EMR would enable patients to freely move between care settings with no loss of documentation and reduce—on all parties—the burden of recall.”	Health system or practice
5.2: Use of shared e-care plans	“The Pharmacists’ Patient Care Process and Pharmacist eCare Plan enable pharmacists’ engagement in MCC to deliver person-centered care and improve health outcomes, while supporting and empowering people at risk for or living with MCC to manage their health and initiate behavioral changes.”	Professional association
	“The eCare Plan is a method under development that uses electronic tools to gather data from each clinician as the patient visits them, allowing a primary care physician access to all relevant opinions, and also to link relevant data to the patient’s own authored goals.”	Industry
5.3: Interoperability and data integration	“Differences in IT tech systems used across the healthcare industry impede functional interoperability and clinicians’ ability to access and consume, i.e., utilize important health data when determining a diagnosis and course of treatment.”	Health system or practice
	“Currently, children with MCC receive care across multiple health systems with electronic health records that do not communicate with each other…The Academy notes that FHIR is probably the best standard, but a barrier to proper integration is that Application Program Interfaces (APIs) still need to be built and integrated across various technical platforms if the patient is receiving care at multiple sites, and there are significant costs associated with interfacing and integration. For example, if the patient is using a popular diabetes app for self-care, an API needs to be created to interface with the hospital’s EHR, and with the primary care provider’s (PCP’s) EHR (assuming these are two different EHRs).”^a^	Professional association
5.4: Patient and caregiver access to EHR data	“Patient portals are now in the best place for communicating with families, but improvements are still needed to ensure interoperability between portals. In addition, electronic consultations can improve subspecialty accessibility for patients with MCC.”	Professional association
5.5: Evidence-informed decision tools	“[Our organization] also supports proactively reframing clinical decision-making tools using valid, evidence-informed measures that incorporate the social determinants of health. It is essential to formally apply an equity lens to the development and reconsideration of all clinical decision-making tools, including clinical practice guidelines, clinical reports, policy statements, and technical reports.”	Professional association
	“AHRQ should take steps to integrate healthcare and social service systems. First, recognize the importance of comprehensive identification of all patient needs, both in the social and health context. Second, patient-centered care tools and analytics should be routinely incorporated so that risk-based assessments can be used to impact care decisions.”	Nonprofit quality improvement, support, or advocacy organization
5.6: Remote digital monitoring	“Particularly for individuals who are managing multiple complex diagnoses and chronic disease, [digital medicine] programs are facilitating access to care and improving the patient experience by allowing them to receive the care they need, when and where they need it—principally through wearable technologies, remote patient monitoring, and virtual provider visits.”^a^	Health system or practice
**Theme 6: Workflow**		
6.1: Redesigning workflow to enable person-centered care for patients with MCC	“Providers’ time would be better spent synthesizing the available information from one centralized site, adapting workflows as appropriate and focusing on delivering the highest quality care to patients.”^a^	Health system or practice
6.2: Workflow enables dynamic comprehensive shared care planning	“The care management team developed a care management tracking system to operationalize key parts of systematic chronic illness care, such as proactive monitoring of outcomes, alerts for patients lost to follow-up, and highlighting patient[s] who had not achieved their target goals. Every week, care managers captured the most current patient outcomes. A weekly clinical summary sheet displayed the illness parameters for each patient.”	Nonprofit quality improvement, support, or advocacy organization
6.3: Using technology to foster collaboration (shared care)	“…we need to remove barriers that prevent coordination and data exchange between behavioral health and primary care. Many patients with multiple chronic conditions—especially among those who are experiencing homelessness or justice-involvement—have mental health and substance use comorbidities that affect their health and social circumstances. However, without timely, bidirectional data sharing between behavioral health providers (in both jails and in the community) and primary care providers, we fall short of our promise to deliver the best possible care for our patients.”^a^	Health system or practice
6.4: Time pressures	“PCPs do not have enough time to provide the guideline-recommended primary care.”	Professional association
6.5: Hybrid (in-person and virtual) care delivery models to engage patients	“[We] encourage AHRQ to support efforts that make hybrid approaches to care delivery readily available to providers and patients. For example, care plans for MCC can be even more effective if providers are able to deploy telehealth, e-consults, and homebased models of care, including urgent care, paramedic services, emergency care, and hospital care.”^a^	Health system or practice and payer
6.6: Risk stratification	“Risk stratification is a valuable strategy to identify and classify patients based on their health status, socioeconomic determinants of health, risk factors, and anticipated health care needs, in order to proactively manage the patient population and resources... As we move towards person-centered, whole person care, policymakers should encourage providers to proactively adopt risk stratification methods to better address the needs of their patients.”^a^	Health system or practice
**Theme 7: Education and self-management support**		
7.1: Increase patient knowledge about the role and benefits of comprehensive care planning	“The final barrier that [this health system] has come across is educating patients on what a comprehensive care model looks like and how we can play a role in helping them manage their chronic conditions.”	Health system or practice
7.2: Patient and caregiver education	“These workshops encourage independence for everyday self-care while, at the same time, reminding participants to talk to their health care providers honestly, i.e. why they aren’t taking their medication as prescribed, if they have fallen, and keep pain/diet journals. A weekly action plan to do something they want to do is requested each week and reported on the following week. This action plan is encouraged, but not required, thus they need to choose to do something and decide for themselves what they want to do.”^a^	Health educator
7.3: Clinician education	“I believe when it comes to our most [vulnerable] populations more needs to be done to educate physicians and nurses in the areas of complex medical concerns, specific syndromes, and aging concerning people with I/DD. You can’t provide person centered goals of care, and a plan that prioritizes benefits and minimizes harm without the proper education and training.”	Clinician
7.4: Empowering patients to self-manage and self-advocate	“[Self-management support] is a short-term intervention to increase the patient’s confidence and ability to take action steps that will address their needs and concerns and improve their health and wellness on their own after your work together is done.”	Clinician and researcher/academic
**Theme 8: Payment**		
8.1: Address insufficient and misaligned payment	“There remains significant need and opportunity to align payment approaches, including related to care management, across payers. A common challenge to sustaining innovative models of care is differences in services coverage or approaches to payment across payers.”	Nonprofit quality improvement, support, or advocacy organization
8.2: Increase value by improving quality and outcomes	“Pay-for-performance programs… provide supplemental payments in conjunction with traditional fee-for-service payments if specified quality, process, or other performance goals are met. Under shared-savings arrangements, participating providers or health systems share in a percentage of net savings (or losses) realized over the course of a given performance year.”^a^	Payer
8.3: Adopt value-based payment models	“To keep the momentum going and enable other organizations to start thinking about caring for patients in this way, the Medicare payment system must shift from rewarding for the volume of care provided in fee-for-service to rewarding for the value of care provided.”	Health system or practice
8.4: Pay for interdisciplinary teams	“In my experience, I see wellness and chronic disease improve when the patient has a [staff member with an] RN (or MSN) [degree as a] Clinical Nurse Manager (Care Manager) who teams with the patient to coordinate care, problem solve, act proactively, educate the patient on self-care, and support or holds patient accountable. However, this is all overhead cost. And for family practice sites, which do not tend to make a profit, the cost of employing such staff can be prohibitive. We need to find a way to make this care financially practical. The current set up, where a small fee can be charged for CM services for care supervised by [an staff member with an] MD [degree], is insufficient.”	Clinician
8.5: Fund integrated person-centered care models	“First, we need greatly increased funding for specialized care models. Current reimbursement schemes are unable to account for the additional expense associated with longer visits, smaller provider panel sizes, and multidisciplinary teams who can coordinate care and engage patients in-between office visits. We need new value-based payment arrangements, such as contracts that allow providers to take risk for total cost of care while also providing adequate adjustments to account for the increased medical and social complexity for disadvantaged populations. Building these payment structures requires concerted and ongoing coordination across city, state, and federal levels of government.”^a^	Health system or practice
8.6: Eliminate out-of-pocket costs for care planning and management services	“In addition, while [this health system] appreciates having a separate code to allow for chronic care management, the cost sharing (copay & unmet deductibles) associated with the visit can be a barrier to patient acceptance and enrollment, especially with populations who have financial struggles. CMS should consider eliminating the cost sharing associated with preventative visits like chronic care management to address the accessibility issues we are facing today.”	Health system or practice
**Theme 9: Achieving community, health system, and payer goals**		
9.1: Address inequities in resource distribution	“One avenue for needed research is using community health centers or FQHCs to address some of these new innovations or models of care…Historically, these innovations have started or imposed on highly resourced systems and are supposed to trickle down; it needs to be the other way around – starting at the community center level.”	Professional association
9.2: Develop meaningful measures that assess quality of person-centered care	“Existing quality measures do not capture what is most important to individuals, particularly older adults with functional limitations and complex health issues.”	Nonprofit quality improvement, support, or advocacy organization
9.3: Harmonize and align measures to drive improvement	“Appropriate quality of care measures that capture what matters to people are urgently needed to improve and incentivize care for the large and growing group of people with complex health needs.”	Nonprofit quality improvement, support, or advocacy organizations
	“We need person-centered quality measures and payment models that recognize and reward longitudinal improvement as opposed to short-term return on investment utilization measures.”	Nonprofit quality improvement, support, or advocacy organizations
9.4: Demonstrate and document return on investment	“From a systems perspective, these programs pay for themselves with cost savings in terms of lower ER and hospital service utilization for patients connected to care, treatment and therapies.”	Clinician and researcher/academic
	“PCSN practices have achieved a 57% reduction in ED visits, 67% reduction in inpatient admissions, improved chronic disease outcomes, and dozens of patients placed in permanent housing.”	Health system or practice

Abbreviations: AHRQ, Agency for Healthcare Research and Quality; app, application; CHW, community health worker; CM, care manager; ED, emergency department; EHR, electronic health record; ER, emergency room; FQHC, Federally Qualified Health Center; HRSN, health-related social need; IDT, interdisciplinary team; I/DD, intellectual and developmental disabilities; IT, information technology; K, kindergarten; MCC multiple chronic conditions; PC, primary care; PCP, primary care practitioner; PCSN, Primary Care Safety Net; SC, specialty care; SDOH, social determinants of health.

^a^ In the context of the survey, provider indicates health care practitioner.

### Theme 1: Suboptimal Quality of Care

Respondents expressed concern about the quality of care provided. Individuals living with MCC and family members described difficulties accessing needed services, including specialty consultations and long-term care services and supports. Some respondents reported being treated unfairly by health care practitioners or receiving inadequate treatment. Clinicians and health care practitioners identified systemic issues within the US health care system—particularly care fragmentation across specialties and settings, limited community-based resources to address social determinants of health, inadequate practice improvement support for clinicians, and delays in implementation of evidence-based practices—limiting care options and negatively impacting the comprehensiveness of care. Respondents pointed to suboptimal health outcomes, avoidable hospitalizations, and preventable health care costs as major consequences of the current state of care and primary motivators for pursuing PCCP. Respondents largely viewed PCCP as a tool for improving quality of care for people living with MCC (Table 2).

### Theme 2: Person-Centered, Goal-Concordant Care

Respondents expressed the need to shift from problem-oriented to person-centered care to achieve better MCC outcomes and described characteristics of person-centered approaches. Respondents across stakeholder groups identified goal concordance as a core characteristic: care should be tailored to the specific needs and goals of the patient and their families. Shared decision-making, through which clinicians share information and make clinical decisions collaboratively with patients and their caregivers, was articulated as integral to goal concordance. Respondents emphasized the importance of meeting patients where they are, both physically and figuratively, to facilitate access to information and services. Whenever possible, strategies for providing information and services should prioritize patient accessibility over system convenience (Table 2).

Respondents overwhelmingly expressed the need for systemic cultural change within the health care system to support the implementation of person-centered, goal-concordant care. They highlighted the importance of addressing social needs, describing how financial stability, secure housing, and social connectedness improve chronic disease management and increase value. Expanded access to effective home- and community-based services is needed. They stressed that language and cultural differences should be recognized and accommodated by care team members in a way that centers patients and families. Respondents expressed the importance of strengthening the evidence base to support clinical decision-making for care planning (Table 2).

### Theme 3: Multidisciplinary Team–Based Care and Care Coordination

Respondents described multidisciplinary team–based care and care coordination as central to PCCP. They noted that person-centered care approaches require investment in the health care workforce to increase numbers and practitioner education across care disciplines in needed competencies. Respondents stressed the importance of including individuals with lived experience on the care team. Health care workers from the same communities as their patients can share valuable experiential knowledge of local community resources. They emphasized that patients and caregivers should be treated as essential members of the multidisciplinary team and collaborate with clinical team members as active care partners (Table 2).

Respondents expressed that behavioral health integration and collaboration across specialties are necessary to effectively coordinate care across multidisciplinary teams. Facilitating linkages to social and community services is critically important. Respondents identified these areas of care coordination as vital for maintaining continuity and improving quality of care during transitions across settings and levels of care (Table 2).

### Theme 4: Prevention Across the Life Course

Respondents emphasized the importance of preventing MCC across the life course, including primary, secondary, and tertiary prevention, and the importance of preventing functional decline among those living with MCC. Investment in prevention effort provides value through improvement in health and functional status. Respondents noted that prevention strategies help optimize functional status by enhancing existing skills and/or modifying the environment. Transitioning from the current disease-centered focus to one that centers patients’ goals was deemed essential to personalizing prevention (Table 2).

### Theme 5: Digital Health Solutions

Respondents highlighted the role of technology in personalizing care and the importance of prioritizing user experience in the development of digital tools. Many stressed that care plans including critical patient information should reside in interoperable and integrated systems, which can be accomplished using shared electronic care plans. Furthermore, patients and caregivers should be able to easily access patients’ health data. Clinicians and researchers relayed the value of evidence-informed decision tools available at the point of care delivery to address the complexity of care planning and the need for evidence to support decision-making. Respondents from health systems reported that remote digital monitoring has revolutionized their delivery of person-centered MCC care, facilitating access and improving patient experience (Table 2).

### Theme 6: Workflow

Respondents expressed frustration with existing care delivery workflows. They recommended redesigning or replacing current tools, practices, and technology to better enable person-centered and comprehensive shared care planning. They underscored the time pressures clinicians face in traditional care delivery models and the increased efficiency of adapting workflows to more seamlessly centralize and track health information related to patient goals. Respondents recommended digital health solutions, including implementing hybrid in-person and virtual care delivery that engages patients at home via telehealth. Interoperability of patient data across care team members and between behavioral and primary care practitioners was underscored as essential. Data sharing between external health care and social service practitioners was acknowledged as challenging but necessary. The value of routinely assessing patients’ health and social risk and using risk stratification to equitably and efficiently allocate clinician time and resources among patients was emphasized (Table 2).

### Theme 7: Education and Self-Management Support

Respondents reported the need to educate clinicians, patients, and caregivers in PCCP and the need for patients and caregivers to understand the role and benefits of comprehensive care planning. Many expressed the need for robust clinician education about the role of PCCP and how to deliver person-centered care. Others highlighted the unique challenges and need for education for those living with and caring for individuals with intellectual and developmental disabilities. Patient empowerment and self-management support were described as essential components of sustainable solutions (Table 2).

### Theme 8: Payment

Respondents underscored that current payment models conflict with patient and clinician goals, presenting a major barrier to widespread adoption of PCCP. Addressing insufficient payment by assuring adequate and aligned funding could increase value by improving quality of care and outcomes. Evidence for the effectiveness of value-based payment models, such as pay-for-performance programs or shared-savings arrangements that incentivize value over volume of care provided, is needed and could foster wider adoption of PCCP. Respondents indicated that multidisciplinary team members, including care coordinators, clinical pharmacists, and community health workers, are not adequately compensated in current payment models. They described inadequate payment as a pervasive financial barrier to adopting PCCP within primary care practices, especially among small independent practices. Respondents identified integrated PCCP models as promising models for sustainably funding longitudinal PCCP. Some recommended out-of-pocket costs for care management services be eliminated to reduce patient-facing financial barriers to care (Table 2).

### Theme 9: Achieving Community, Health System, and Payer Goals

Respondents suggested several approaches for achieving stakeholder goals to successfully implement PCCP within primary care and other ambulatory settings, including developing innovative models in lower-resourced settings, including community health centers, rather than adapting models from highly resourced systems. Meaningful person-centered quality metrics that prioritize outcomes that matter most to people living with MCC, especially longitudinal outcomes as opposed to short-term utilization measures, were communicated as key to incentivizing efforts to drive PCCP implementation. Respondents expressed the need for person-centered models to capture data to continuously improve person-centered care delivery and document return on investment (Table 2).

## Discussion

This qualitative study identified 9 themes for strategies for, as well as facilitators and barriers to implementation of PCCP: (1) suboptimal quality of care; (2) person-centered, goal-concordant care; (3) multidisciplinary team–based care and care coordination; (4) prevention across the life course; (5) digital health solutions; (6) workflow; (7) education and self-management support; (8) payment; and (9) achieving community, health system, and payer goals. These themes identified reforms needed and components of care delivery models to support PCCP. The many barriers to implementing PCCP include time constraints, inadequate payment, and workforce availability and competencies. Promising solutions were identified including, multidisciplinary teams, digital health technologies, workflow changes, payment policies, goal alignment, and opportunities for aligning practice, policy, and payment to advance PCCP. Understanding how to effectively provide comprehensive, person-centered care is foundational to improving health outcomes for people living with or at risk for MCC and increasing value in care delivery. AHRQ’s RFI elicited robust responses from diverse health care sectors, researchers, clinicians, community organizations, patients, and caregivers who consistently articulated that traditional models for delivering care are not effective in optimizing health and functioning.

Respondents and stakeholder groups used varied terminology, theoretical frameworks, and references to define PCCP from their position on the care team or their discipline. Despite differences, there was substantial thematic overlap across responses. Respondents emphasized the importance of holistic, goal-concordant, integrated, and team-based care; how such care is supported by processes and technologies that facilitate communication and data sharing across the care team; and how such care is sustained through value-based payment models. These themes align with evidence-based models for providing care to people with MCC and/or complex care needs, reflecting the prioritization of patient goals in goal-oriented care,^26^ collaborative communication and data sharing between clinicians and patients central to shared decision-making,^27,28,29^ and comprehensive coordination of medical and social services in patient-centered medical homes.^30^

Coordination and communication across care teams are essential to addressing a patient’s multiple, complex, and sometimes contradicting needs.^31,32^ For example, a person with heart failure may be told by a cardiologist to restrict fluids, but by a nephrologist to assure adequate hydration. A patient with Parkinson disease and their care partners may need to weigh the tradeoffs of a medication change that improves mobility but exacerbates dementia behavioral symptoms. This can be confusing and distressing to patients and their caregivers. Recommendations are not always made with patient’s goals at the center. The implementation of the 4M’s (What Matters, Medication, Mentation, Mobility) framework for age-friendly health systems aligns with PCCP, centering patient’s goals and emphasizing that clinical decisions require consideration of what matters most to the patient.^33^ Patient Priorities Care has developed tools to facilitate goal identification by interdisciplinary teams to foster PCCP.^34,35^ Practice and policy changes to deliver PCCP can benefit health systems users by supporting the caring function of health care.^36^

To our knowledge, this is the first report on the role of PCCP, solutions for care delivery, and facilitators and barriers to implementation from diverse disciplinary and geographic perspectives. This analysis was undertaken to inform AHRQ’s efforts toward building the evidence base for more widespread adoption and scale of PCCP as a component of routine practice.^37^ This priority emerged from extensive consultations and a 2020 summit where PCCP was identified as a lynchpin for care transformation to improve MCC care delivery.^38^ The summit led to AHRQ’s MCC research agenda^37^ and publications on PCCP in learning health systems,^39^ digital health solutions,^40^ and patient and family engagement.^41^ The results of these analyses are informing AHRQ’s priority setting, strategic planning, and funding announcement development. The Person-Centered Care Planning for Persons With Multiple Chronic Conditions initiative aims to identify innovative, feasible models of PCCP through national engagement of health system leaders, professional associations, policymakers, and a learning community of frontline health care staff, implementers, and researchers.^42,43^ A 2023 roundtable of AHRQ partners from multiple sectors and disciplines identified many related efforts under way, albeit siloed, to improve MCC care and the enormous potential in aligning these efforts and shared learning.^44^ AHRQ, together with the National Institute of Diabetes and Digestive and Kidney Diseases, developed an interoperable electronic care plan to support longitudinal comprehensive shared care planning, including clinician- and patient- and caregiver-facing Substitutable Medical Applications and Reusable Technologies on FHIR applications to facilitate PCCP, data aggregation across multiple electronic health records, and collection of data on health-related social needs, functional status, patient-reported outcomes, and goals.^45,46^ This work advances Department of Health and Human Services and Biden Administration priorities of integrating health and social care and addressing the social determinants of health.^47,48,49^

### Limitations

This study has some limitations. Respondents’ perspectives may not be representative of people lacking the technological access and time necessary to monitor and respond to the RFI. AHRQ received fewer and, on average, shorter responses from individual patients, family members, and caregivers than from clinicians, researchers, and organizations; thus, the perspectives from this important stakeholder group are comparatively underrepresented. However, the RFI was widely circulated through federal partners, networks, and social media and received a robust response. We were unable to probe responses. However, respondents were encouraged to provide all information they felt relevant, even if not directly solicited by the questions.

## Conclusions

This qualitative study of individuals and organizations across the health care system found that challenges in advancing PCCP are substantial and require aligning payment, policy, culture change, along with evidence for models of care and implementation strategies for successful adoption. PCCP is central to health care redesign to deliver person-centered care. Insights gained from this analysis are informing research priorities, dissemination, and implementation efforts to transform care for people living with and at risk for MCC.

## Notes

Supplement.
Data Sharing Statement

## References

[1] Skou ST, Mair FS, Fortin M, . Multimorbidity. Nat Rev Dis Primers. 2022;8(1):48. doi:10.1038/s41572-022-00376-4 35835758 PMC7613517

[2] Vogeli C, Shields AE, Lee TA, . Multiple chronic conditions: prevalence, health consequences, and implications for quality, care management, and costs. J Gen Intern Med. 2007;22(Suppl 3)(suppl 3):391-395. doi:10.1007/s11606-007-0322-1 18026807 PMC2150598

[3] Rathert C, Wyrwich MD, Boren SA. Patient-centered care and outcomes: a systematic review of the literature. Med Care Res Rev. 2013;70(4):351-379. doi:10.1177/1077558712465774 23169897

[4] Baker A, Cronin K, Conway P, DeSalvo K, Rajkumar R, Press M. Making the comprehensive shared care plan a reality. NEJM Catal. Published online May 16, 2016. doi:10.1056/CAT.16.0838

[5] Kogan AC, Wilber K, Mosqueda L. Person-centered care for older adults with chronic conditions and functional impairment: a systematic literature review. J Am Geriatr Soc. 2016;64(1):e1-e7. doi:10.1111/jgs.13873 26626408

[6] Burt J, Rick J, Blakeman T, Protheroe J, Roland M, Bower P. Care plans and care planning in long-term conditions: a conceptual model. Prim Health Care Res Dev. 2014;15(4):342-354. doi:10.1017/S1463423613000327 23883621 PMC3976966

[7] Tinetti M, Dindo L, Smith CD, . Challenges and strategies in patients’ health priorities-aligned decision-making for older adults with multiple chronic conditions. PLoS One. 2019;14(6):e0218249. doi:10.1371/journal.pone.0218249 31181117 PMC6557523

[8] Tinetti ME, Naik AD, Dindo L, . Association of patient priorities-aligned decision-making with patient outcomes and ambulatory health care burden among older adults with multiple chronic conditions: a nonrandomized clinical trial. JAMA Intern Med. 2019;179(12):1688-1697. doi:10.1001/jamainternmed.2019.4235 31589281 PMC6784811

[9] Edmonds AT, Rhew IC, Jones-Smith J, Chan KCG, Nelson K, Williams EC. Patient-centered primary care and receipt of evidence-based alcohol-related care in the national Veterans Health Administration. J Subst Abuse Treat. 2022;138:108709. doi:10.1016/j.jsat.2021.108709 35277305

[10] Liang H, Zhu J, Kong X, Beydoun MA, Wenzel JA, Shi L. The patient-centered care and receipt of preventive services among older adults with chronic diseases: a nationwide cross-sectional study. Inquiry. 2017;54:46958017724003. 28814174 10.1177/0046958017724003 PMC5798736

[11] Tavares J, Cohen M, Hwang A, Hawes FM. Person-centered care: why taking individuals’ care preferences into account matters. Accessed May 14, 2024. https://communitycatalyst.org/wp-content/uploads/2023/09/Person-Centered-Care-Report-Why-it-Matters.pdf

[12] Berntsen GKR, Dalbakk M, Hurley JS, . Person-centred, integrated and pro-active care for multi-morbid elderly with advanced care needs: a propensity score-matched controlled trial. BMC Health Serv Res. 2019;19(1):682. doi:10.1186/s12913-019-4397-2 31581947 PMC6777026

[13] Berry LL, Rock BL, Smith Houskamp B, Brueggeman J, Tucker L. Care coordination for patients with complex health profiles in inpatient and outpatient settings. Mayo Clin Proc. 2013;88(2):184-194. doi:10.1016/j.mayocp.2012.10.016 23290738

[14] Social Security Administration. Compilation of the Social Security laws: provisions respecting inapplicability and waiver of certain requirements of this title. Accessed December 4, 2023. https://www.ssa.gov/OP_Home/ssact/title19/1915.htm

[15] Social Security Administration. Compilation of the Social Security laws: requirements for, and assuring quality of care in, skilled nursing facilities. Accessed December 4, 2023. https://www.ssa.gov/OP_Home/ssact/title18/1819.htm

[16] Fowler E, Fogler S, Schreiber C, . The CMS Innovation Center’s strategy to support person-centered, value-based specialty care: 2024 update. *Health Affairs Forefront*. April 2, 2024. Accessed May 14, 2024. https://www.healthaffairs.org/content/forefront/cms-innovation-center-s-strategy-support-person-centered-value-based-specialty-care

[17] Donohue JM, Cole ES, James CV, Jarlenski M, Michener JD, Roberts ET. The US Medicaid Program: coverage, financing, reforms, and implications for health equity. JAMA. 2022;328(11):1085-1099. doi:10.1001/jama.2022.14791 36125468

[18] Chen AH, Ghaly MA. Medicaid as a driver of health equity. JAMA. 2022;328(11):1051-1052. doi:10.1001/jama.2022.14911 36125484

[19] American Geriatrics Society Expert Panel on Person-Centered Care. Person-centered care: a definition and essential elements. J Am Geriatr Soc. 2016;64(1):15-18. doi:10.1111/jgs.13866 26626262

[20] US National Archives. Request for information on person-centered care planning for multiple chronic conditions (MCC). Accessed May 14, 2024. https://www.federalregister.gov/documents/2022/09/16/2022-20027/request-for-information-on-person-centered-care-planning-for-multiple-chronic-conditions-mcc

[21] Braun V, Clarke V. Using thematic analysis in psychology. Qual Res Psychol. 2006;3(2):77-101. doi:10.1191/1478088706qp063oa

[22] Braun V, Clarke V. What can “thematic analysis” offer health and wellbeing researchers? Int J Qual Stud Health Well-being. 2014;9(1):26152. doi:10.3402/qhw.v9.26152 25326092 PMC4201665

[23] Braun V, Clarke V. One size fits all: what counts as quality practice in (reflexive) thematic analysis? Qual Res Psychol. 2021;18(3):328-352. doi:10.1080/14780887.2020.1769238

[24] Jain A, Brooks JR, Alford CC, . Awareness of racial and ethnic bias and potential solutions to address bias with use of health care algorithms. JAMA Health Forum. 2023;4(6):e231197-e231197. doi:10.1001/jamahealthforum.2023.1197 37266959 PMC10238944

[25] Watkins DC. Rapid and rigorous qualitative data analysis:the “RADaR” technique for applied research. Int J Qual Methods. Published online June 8, 2017. doi:10.1177/1609406917712131

[26] Steele Gray C, Grudniewicz A, Armas A, Mold J, Im J, Boeckxstaens P. Goal-oriented care: a catalyst for person-centred system integration. Int J Integr Care. 2020;20(4):8. doi:10.5334/ijic.5520 33199976 PMC7646288

[27] Barry MJ, Edgman-Levitan S. Shared decision making—pinnacle of patient-centered care. N Engl J Med. 2012;366(9):780-781. doi:10.1056/NEJMp1109283 22375967

[28] Tonelli MR, Sullivan MD. Person-centred shared decision making. J Eval Clin Pract. 2019;25(6):1057-1062. doi:10.1111/jep.13260 31407417

[29] Verberne WR, Stiggelbout AM, Bos WJW, van Delden JJM. Asking the right questions: towards a person-centered conception of shared decision-making regarding treatment of advanced chronic kidney disease in older patients. BMC Med Ethics. 2022;23(1):47. doi:10.1186/s12910-022-00784-x 35477488 PMC9047263

[30] Mold JW, Duffy FD. How patient-centered medical homes can bring meaning to health care: a call for person-centered care. Ann Fam Med. 2022;20(4):353-356. doi:10.1370/afm.2827 35879079 PMC9328711

[31] Bodenheimer T, Wagner EH, Grumbach K. Improving primary care for patients with chronic illness. JAMA. 2002;288(14):1775-1779. doi:10.1001/jama.288.14.1775 12365965

[32] Wagner EH, Austin BT, Davis C, Hindmarsh M, Schaefer J, Bonomi A. Improving chronic illness care: translating evidence into action. Health Aff (Millwood). 2001;20(6):64-78. doi:10.1377/hlthaff.20.6.64 11816692

[33] Mate KS, Berman A, Laderman M, Kabcenell A, Fulmer T. Creating age-friendly health systems—a vision for better care of older adults. Healthc (Amst). 2018;6(1):4-6. doi:10.1016/j.hjdsi.2017.05.005 28774720

[34] Tinetti ME, Costello DM, Naik AD, . Outcome goals and health care preferences of older adults with multiple chronic conditions. JAMA Netw Open. 2021;4(3):e211271-e211271. doi:10.1001/jamanetworkopen.2021.1271 33760091 PMC7991967

[35] Tinetti ME, Naik A. Patient priorities care. Accessed May 14, 2024. https://patientprioritiescare.org/

[36] Montori VM, Hargraves I, McNellis RJ, . The Care and Learn Model: a Practice and Research Model for Improving Healthcare Quality and Outcomes. J Gen Intern Med. 2019;34(1):154-158. doi:10.1007/s11606-018-4737-7 30430403 PMC6318165

[37] Bierman AS, Wang J, O’Malley PG, Moss DK. Transforming care for people with multiple chronic conditions: Agency for Healthcare Research and Quality’s research agenda. Health Serv Res. 2021;56(Suppl 1)(suppl 1):973-979. doi:10.1111/1475-6773.13863 34378192 PMC8515222

[38] Agency for Healthcare Research and Quality. Proceedings from AHRQ Summit on Transforming Care for People Living with Multiple Chronic Conditions 2020. Accessed May 14, 2024. https://www.ahrq.gov/sites/default/files/wysiwyg/patient-safety/settings/mcc-summit/mcc-summit-proceedings.pdf

[39] Bierman AS, Mistry KB. Commentary: achieving health equity—the role of learning health systems. Healthc Policy. 2023;19(2):21-27. doi:10.12927/hcpol.2023.27236 38105664 PMC10751753

[40] Samal L, Fu HN, Camara DS, Wang J, Bierman AS, Dorr DA. Health information technology to improve care for people with multiple chronic conditions. Health Serv Res. 2021;56(Suppl 1)(suppl 1):1006-1036. doi:10.1111/1475-6773.13860 34363220 PMC8515226

[41] Vick JB, Wolff JL. A scoping review of person and family engagement in the context of multiple chronic conditions. Health Serv Res. 2021;56(Suppl 1)(suppl 1):990-1005. doi:10.1111/1475-6773.13857 34363217 PMC8515220

[42] Bierman AS, Werner RM, eds. The science of care for people with multiple chronic conditions. Health Serv Res. 2021;56(S1):965-1079. doi:10.1111/1475-6773.13863 PMC8515226 34363220

[43] Agency for Healthcare Research and Quality. Advancing patient-centered care for people living with multiple chronic conditions. Accessed May 14, 2024. https://www.ahrq.gov/patient-safety/settings/long-term-care/resource/multichronic/mcc.html

[44] Weinick RM, Carney MT, Milnes T, Bierman AS. Optimizing health and function as we age roundtable report. Accessed May 14, 2024. https://www.ahrq.gov/sites/default/files/wysiwyg/ncepcr/tools/healthy-aging-roundtable.pdf

[45] Norton JM, Ip A, Ruggiano N, . Assessing progress toward the vision of a comprehensive, shared electronic care plan: scoping review. J Med Internet Res. 2022;24(6):e36569. doi:10.2196/36569 35687382 PMC9233246

[46] Agency for Healthcare Research and Quality. Multiple chronic conditions e-Care Plan project. Accessed July 7, 2023. https://ecareplan.ahrq.gov/

[47] Chappel A, Cronin K, Kulinski K, . Improving health and well-being through community care hubs. *Health Affairs Forefront*. November 29, 2022. Accessed May 14, 2024. https://www.healthaffairs.org/content/forefront/improving-health-and-well-being-through-community-care-hubs

[48] Domestic Policy Council; Office of Science and Technology Policy. The U.S. playbook to address social determinants of health. Accessed May 14, 2024. https://www.whitehouse.gov/wp-content/uploads/2023/11/SDOH-Playbook-4.pdf

[49] US Department of Health and Human Services. Call to action: addressing health-related social needs in communities across the nation. Accessed May 14, 2024. https://aspe.hhs.gov/sites/default/files/documents/3e2f6140d0087435cc6832bf8cf32618/hhs-call-to-action-health-related-social-needs.pdf

