# Supplementary material for: Person-Centered Care Planning for People Living With or at Risk for Multiple Chronic Conditions
Source: JAMA Netw Open. 2024 Oct 17;7(10):e2439851. doi: 10.1001/jamanetworkopen.2024.39851 (PMC11581598; doi:10.1001/jamanetworkopen.2024.39851)
Supplement: Supplement. — Data Sharing Statement [file jamanetwopen-e2439851-s001.pdf]

Data Sharing Statement

Watson. Person-Centered Care Planning for People Living With or at Risk for Multiple Chronic Conditions. *JAMA Netw Open*. Published October 17, 2024.
doi:10.1001/jamanetworkopen.2024.39851

Data
Data available: No

Additional Information
Explanation for why data not available: This would be published as a Special Communication.
